# A Scale for Assessing Pain in Older Adults: The Influence on Activities of Daily Living

**DOI:** 10.3390/healthcare13060660

**Published:** 2025-03-17

**Authors:** Zuleyma González Miguel, María de Lourdes García Hernández, Yolanda Hernández Ortega, María Alberta García Jiménez, María de Lourdes Rico González, Patricia Cruz Bello, María Gicela Pérez Hernández, Nicolas Santiago-González, Marisol Ignacio Albino, Erika López Martiñón

**Affiliations:** 1 Faculty of Dentistry, Autonomous University of the State of Mexico, Toluca 50200, Mexico; zgonzalezm@uaemex.mx; 2 Faculty of Nursing and Obstetrics, Autonomous University of the State of Mexico, Toluca 50200, Mexico; yhernandezo@uaemex.mx (Y.H.O.); mlricog@uaemex.mx (M.d.L.R.G.); pcruzb@uaemex.mx (P.C.B.); mignacioa@uaemex.mx (M.I.A.); elopezma@uaemex.mx (E.L.M.); 3 Faculty of Nursing, Autonomous Metropolitan University, Mexico City 1100, Mexico; ajimenez@correo.xoc.uam.mx; 4 Faculty of Nursing, University of Colima, Colima 28040, Mexico; ggise_ph@ucol.mx; 5 Nursing Research Projects Unit, Regional Hospital of High Speciality of Ixtapaluca, Health Services of the Mexican Institute of Social Security for Welfare (IMSS-BIENESTAR), Ixtapaluca 56530, Mexico; nicosantiago21@hotmail.com

**Keywords:** evaluation scale, pain, seniors, activities of daily living

## Abstract

Physical functional impairment generates pain, fear and anguish, impacting the activities of daily living (ADLs) of older adults (OAs); The background was to design an instrument that can measure pain in OA living with pain. **Methods:** mixed analytical and cross-sectional study in two non-probabilistic samples: 15 experts in the care of OAs and 185 OAs from day homes in three municipalities of the State of Mexico. The instruments used included an integrated scale of 30 items, elaborated with the Delphi technique over three rounds, and the Scale for Measuring Pain in Older Adults (SMPOA), with 30 items divided into four factors. An analysis was conducted using content validity ratios and the Lawshe´s method adapted by Tristán for qualitative studies, with a minimum of 0.87 and a maximum of 1.0. An exploratory factor analysis (EFA) was conducted to structure the underlying factors into a data matrix, addressing the relationships between a set of variables, and a confirmatory factor analysis (CFA) was conducted to validate the scale using the main axis method and rotation via Quartimax with Kaiser normalisation. **Results:** proposal of a scale to measure pain in MA (EMEDAM). **Conclusion:** the adapted scale assesses sensory and affective intensity and the influence on the ADLs and can be used in OAs living with acute or chronic pain.

## 1. Introduction

Pain is defined as an unpleasant sensory and emotional experience associated with tissue damage [1]. Williams and Craig defined pain as a distressing experience associated with actual or potential tissue damage, with sensory, emotional, cognitive, and social components [2]. The prevalence of pain in older adults (OAs) ranges from 25% to 88% and increases with age; 6.7% of women suffer from pain [3]. Pain can emerge from a variety of innervated structures [4], and the pain becomes chronic when it persists for at least three months [5]. In 2018, Corsi showed that pain generates cognitive alterations, a higher level of depression, and less independence in OAs [6]. As a precedent for the assessment of pain using a scale, Keele proposed the first scale to measure pain in 1948; later, in 1972, Woodforde and Merskey presented the Visual Analog Scale (VAS), used by Scott and Huskisson, and then, in 1976, validated the scale in the clinical setting [7]. In 1975, Melzack and Casey created the McGill Pain Questionnaire, and in 1978, Downie created a one-dimensional numerical pain scale [8]. Other scales to assess pain include the West Haven–Yale Multidimensional Pain Inventory (WHYMPI), which arose from the cognitive theory of behavior [9]; the PainDETEC scale, with its theory of neuropathic pain intensity; and the Visual Analog Scale, which is based on the one-dimensional theory of pain [10]. Based on the aforementioned theoretical references and taking into account the biological, physical, psychological, and social characteristics of OAs, in this study, the Scale to Measure Pain in Older Adults (SMPOA) was designed and validated.

The United Nations (UN) established that a person is considered an OA from the age of 60 in developing countries and from the age of 65 in developed countries [11]. On the other hand, the World Health Organization (WHO) characterizes old age based on the presence of geriatric syndromes [12], the most common form of which is frequent diseases. Due to these health conditions, OAs experience systemic deterioration, making them vulnerable [13]. For this reason, the physiological and anatomical changes accompanied by aging lead to a decrease in activities and physiological functions and make OAs vulnerable to developing pain, which can promote the risk of physical and functional dependence. Data from the National Institute of Statistics and Geography (INEGI) in Mexico in 2019 indicate that out of every 100 people with disabilities, 51 are OAs and that difficulty performing the activities of daily living (ADLs) is closely related to increasing age [14] and the presence of pain, resulting in emotional changes and further exacerbating the inability to carry out activities of daily living (ADLs). Alvarado (2016) describes OAs’ experiences with benign chronic pain as a persistent unpleasant sensation that develops over three to six months, generating anguish, sadness, fear, stress, helplessness, hopelessness, and isolation [15]. Likewise, chronic pain can be caused by musculoskeletal diseases (nociceptive pain), some type of neuralgia (neuropathic pain), or a combination of diseases (mixed pain) [16]. Therefore, assessing the characteristics of pain, sensory and affective intensity, and their influences on ADLs is important.

Although scales that measure pain and its intensity exist, none are specifically designed to measure pain in OAs. In Mexico, scales used to evaluate pain and its influence on the affective state and the ADLs of OAs are lacking. However, since OAs are at a stage of life where they are likely to experience biological deterioration; chronic disease; and/or losses of employment, their partner, and their social roles, which generates emotional and affective pain, instruments are required to clinically assess and identify the origin of their pain and its impact on ADLs. The objective of this research was to design a scale to assess pain in older adults and its influence on the activities of daily living, with the specific objective of evaluating pain in a population of OAs who attend a day home in order to statistically evaluate the scale.

## 2. Materials and Methods

This study used a mixed method with two independent samples. To design the SMPOA, a qualitative analysis was conducted by assessing the experiences and opinions of experts using the Delphi technique proposed by Abraham Kaplan in the 1940s, as this method is used to reach a consensus among a group of experts when analyzing and reflecting on a specific problem; by controlling consensus, biases can be minimized [17]. A quantitative, analytical, and cross-sectional analysis was then conducted to statistically validate the scale.

In the first analysis, the sample consisted of n = 15 experts in gerontology, gathered using an intentional non-probabilistic sampling method, with the following inclusion criteria: experts in the clinical care of OAs, with more than three years of experience working in hospitals and health centers, who are aware of the reality and context of OAs with pain, residing in the State of Mexico, willing to participate in this study, willing to make contact with each other in work sessions, and who provided informed consent.

In the second sample from the quantitative portion of this study, the sample was made up of 352 OAs located in 10 day homes in the municipalities of San Mateo Atenco, Zinacantepec, and Toluca in the State of Mexico. The sample was obtained via the non-probabilistic sampling method with a 95% confidence level and 5% margin of error and consisted of 185 OAs.

**Figure 1:**
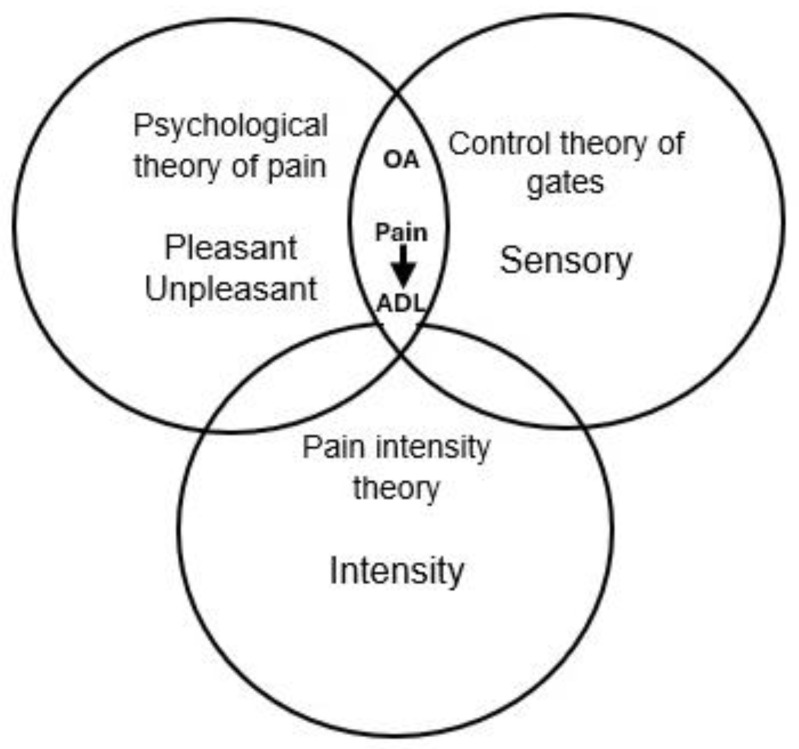
Relationship between pain theories in OAs and their ADLs. Source: model of pain in OAs under pain intensity theory (1874), gate control theory (1965), and psychological theory of pain (1965).

The construction of the instrument was based on the following theories, taking the intensity and perception of pain, and the behavior that pain can induce in OAs into consideration: Erb’s (1874) pain intensity theory, in which any sensory stimulus is capable of causing pain when it reaches sufficient intensity; Melzack and Wall’s (1965) gate control theory, which propose that the perception of pain passes through a series of nerve pathways; the psychological theory of pain, which states that pain is an unpleasant experience [18,19]; one item of the PainDETEC scale; and six of the WHYMPI, as shown in Figure 1.

### Procedures

This study began by designing 26 items based on the aforementioned theories: items 8 and 9 were from the pain intensity theory; items 1, 2, 3, 4, 5, 6, and 7 were from the gate control theory; items 11, 12, 13, 14, and 15 items were from the psychological theory of pain; and 12 items corresponded to the influence of pain on ADLs.

Subsequently, the group of researchers selected experts according to the inclusion criteria, resulting in 15 gerontologists who are experts in the clinical care of OAs being invited, by phone, to participate after being given an explanation of the objective of the research and the procedure. Only those who accepted, signed informed consent, and provided their contact information participated in this study.

The 26-item scale was evaluated across three rounds: in the first round, the experts suggested that 4 items be added to measure the impact of pain on ADLs and the wording of items 4, 6, and 10 (Table 3) be corrected. The results were analyzed, and feedback was given.

The second and third rounds were carried out with the new 30-item scale; the experts made suggestions to correct the wording, clarity, and coherence of the questions in each of the items (Tables 4 and 5).

At the end of the rounds, the group of researchers analyzed the final set of items and proceeded to evaluate each of them using the model of Escobar and Cuervo (2008), which measures four constructs: 1. sufficiency, which identifies whether the question belongs to a single dimension that achieves its objective; 2. clarity, which considered the simplicity and ease of understanding of the question and bias avoidance in potential answers; 3. coherence, which assessed whether the question was logically related to what is being measured; and 4. relevance, which evaluated whether the question was adequate and important for achieving the objective. A category score indicator was used, giving a score from 1 to 4 on a Likert scale, as described in Table 1.

After the evaluation and measurement of each item based on the above constructs, the 30 items were statistically validated with the Lawshe method adapted by Tristan to obtain content validity ratios (CVRs), which evaluate the validity of items of an instrument based on expert opinion and considers whether the question is essential, useful, and/or necessary. Acceptable content validity ratios range from 0.80 to 1.0 [20]. From this validation, the 30 items obtained scores between 0.87 and 1.00; thus, they were considered for the Scale to Measure Pain in Older Adults (SMPOA).

Upon obtaining an acceptable score of 1.00 with the Lawshe method, an exploratory factor analysis (EFA) was carried out to identify the relations between the study variables and to create a matrix of the underlying factors or dimensions of pain. McDonald’s Omega reliability coefficient was determined per factor in the IBM SPSS version 29 program, resulting in four dimensions with a total of 24 items. The final statistical validation of the SMPOA was performed with a confirmatory factor analysis (CFA) to validate the scale via principal axis factorization and Quartimax rotation with Kaiser normalization in a group of OAs (185). The objective of this study was made known, and informed consent was read aloud to authorize participation. Interviews were carried out with each of the participants, with each item and their corresponding answer options read aloud. The interviews took place in the day houses, ensuring that the lighting and environment were comfortable and that distractions and interruptions could be avoided.

This study adhered to the following ethical principles laid out in the Declaration of Helsinki: show respect to people and safeguard their integrity, inform them about the objectives of the study, and allow them free choice to participate. This study was also in compliance with the General Health Law on respect for dignity and protection of rights and well-being. The research was approved by the Ethics and Research Committee of the Autonomous University of the State of Mexico, Faculty of Nursing.

## 3. Results

A total of 15 experts participated in the design of the items, of which 11 (73%) were female and 4 (27%) were male, 15 had a bachelor’s degree in gerontology (100%) and 4 (27%) had postgraduate degrees in an area of health, 2 (13%) worked in a hospital and 13 (87%) worked in health centers, and 7 (47%) had 3 to 5 years of work experience and 8 (53%) had more than 6 years of work experience (Table 2).

The first round of qualitative measurement comprised twenty-six items and four constructs—sufficiency, clarity, coherence, and relevance—evaluated to obtain a maximum content validity ratio (CVR) of 1.0 and a minimum of 0.33. Items below the acceptable CVR were identified; four questions related to the ADLs—bathing, eating, dressing, and going to the bathroom—were added; and the wording of two questions was modified—those related to the sensations of whiplash and numbness—as can be seen in Table 3.

**Table 3:** Proposed items of SMPOA evaluated in first round.

Items	Sufficiency	Clarity	Coherence	Relevance
	CVR’	CVR’	CVR’	CVR’
1. You feel a pulsing sensation when there is pain:	0.80	0.60 *	0.80	0.80
2. You feel cold when you have pain:	0.87	1.00	0.93	0.87
3. You feel warm when you have pain:	0.87	1.00	0.80	0.87
4. You feel the sensation of whiplash when there is pain:	0.73 *	0.33 *	0.60	0.73 *
5. You feel numbness when there is pain:	0.93	0.80	0.80	0.73 *
6. You feel a stabbing sensation when there is pain:	0.80	0.73 *	0.80	0.73 *
7. You feel tense when there is pain:	0.87	1.00	0.80	0.93
8. The pain you feel is deep:	0.80	0.80	0.93	0.87
9. The pain you feel makes you dizzy:	0.87	0.87	0.93	0.80
10. The pain you present is mortifying:	0.67 *	0.53 *	0.87	0.67 *
11. The pain you feel is disabling:	0.93	0.93	0.93	0.93
12. The pain you feel irritates you:	0.87	0.73 *	0.87	0.87
13. The pain you feel depresses you:	0.93	0.80	0.87	0.93
14. The pain you feel makes you desperate:	0.80	0.73 *	0.80	0.67 *
15. To what extent does pain interfere with your daily activities?	1.00	0.93	1.00	1.00
16. Since you started experiencing pain, how much has your ability to perform daily activities changed?	0.93	0.80	1.00	1.00
17. How bad has your pain been over the past week?	0.80	0.80	0.93	0.93
18. How much has your pain changed your ability to participate in recreational and social activities?	1.00	0.80	1.00	1.00
19. How much has your pain changed your ability to perform routine household activities?	0.87	0.73 *	0.87	0.93
20. Take care of the garden	0.80	0.67 *	0.80	0.73 *
21. Go for a walk	0.87	0.73 *	0.73 *	0.73 *
22. Go grocery shopping	0.87	0.73 *	0.73 *	0.73 *
23. Prepare food	0.87	0.73 *	0.73 *	0.67 *
24. Visit friends	0.87	0.67 *	0.73 *	0.73 *
25. Visit family	0.80	0.67 *	0.73 *	0.80
26. Help with house cleaning	0.80	0.67 *	0.73 *	0.73 *

Source: The data on expert opinions obtained with the Delphi technique, 2024. Note: items marked with an asterisk (*) did not reach content validity, and a second round was held.

With the obtained expert opinions, four questions were proposed to be added, giving a total of thirty items, and a second round of expert feedback was carried out, leading to the following results: for the sufficiency, clarity, and coherence constructs, 96.2% (29 items) acceptance was achieved, achieving a minimum CVR of 0.73 and a maximum of 1.0; regarding the relevance category, 3.8% (one item) did not reach validity, as can be seen in Table 4.

**Table 4:** Proposed items of SMPOA evaluated in second round.

Items	Sufficiency	Clarity	Coherence	Relevance
	CVR’	CVR’	CVR’	CVR’
1. You feel a pulsing sensation when there is pain:	1.00	0.80	0.93	1.00
2. You feel cold when you have pain:	1.00	1.00	1.00	1.00
3. You feel warm when you have pain:	0.93	1.00	1.00	0.93
4. You feel a pinching sensation when there is pain:	1.00	0.80	0.93	1.00
5. You feel numbness when there is pain:	0.93	0.93	0.87	0.87
6. You feel a stabbing sensation when there is pain:	0.93	0.80	0.80	0.87
7. You feel tense when there is pain:	0.93	0.87	0.93	1.00
8. The pain you feel is deep:	1.00	1.00	1.00	0.93
9. How bad has your pain been over the past week?	0.93	0.87	0.93	0.93
10. The pain you feel makes you dizzy:	0.87	0.87	0.80	0.80
11. The pain you present is annoying and makes you angry:	0.93	0.87	0.93	0.87
12. The pain you feels causes you anguish:	0.93	0.93	0.93	1.00
13. The pain you feel irritates you:	0.93	0.93	1.00	1.00
14. The pain you feel depresses you:	0.87	0.87	0.93	1.00
15. The pain you feels makes you desperate:	0.87	0.87	0.87	0.93
16. Pain interferes with your daily activities	0.93	0.93	1.00	1.00
17. Pain has changed your ability to perform daily activities:	0.87	0.93	1.00	1.00
18. How much has your pain changed your ability to participate in recreational and social activities?	1.00	0.93	0.93	0.93
19. Pain has changed your ability to perform routine household activities:	0.80	0.87	0.93	0.87
20. Since you started to experience pain, you have still been able to take care of the garden	1.00	0.87	0.87	0.87
21. Since you started to experience pain, you have still been able to go for a walk	0.93	0.87	0.87	0.87
22. Since you started to experience pain, you have still been able to go shopping for food	0.80	0.87	0.80	0.80
23. Since you started to experience pain, you have still been able to prepare food	0.93	0.93	0.87	0.87
24. Since you started to experience pain, you have still been able to visit friends	0.87	0.87	0.80	0.80
25. Since you started to experience pain, you have still been able to visit your family	0.87	0.80	0.87	0.73
26. Since you started to experience pain, you have still been able to help clean the house	0.87	0.87	0.80	0.80
27. Since you started to experience pain, you have still been able to bathe yourself	0.87	0.87	0.80	0.80
28. Since you started to experience pain, you have still been able to eat on your own	0.87	0.87	0.80	0.80
29. Since you started to experience pain, you have still been able to dress yourself	0.93	0.87	0.87	0.80
30. Since you started to experience pain, you have still been able to go to the bathroom on your own	0.93	0.93	0.80	0.80

Source: Data on expert opinions obtained with Delphi technique, 2024.

During the second round, the experts suggested revising the wording of some items, emphasizing item 25 as needing revision; its wording was corrected and submitted for a third round, as shown in Table 5.

**Table 5:** The proposed items of the SMPOA evaluated in the third round.

Items	Sufficiency	Clarity	Coherence	Relevance
	CVR’	CVR’	CVR’	CVR’
1. You feel a pulsing sensation when there is pain:	1.00	1.00	1.00	1.00
2. You feel cold where you have pain:	1.00	1.00	1.00	1.00
3. You feel warm where you have pain:	1.00	1.00	1.00	1.00
4. You feel a pinching sensation when there is pain:	1.00	1.00	1.00	1.00
5. You feel numbness when there is pain:	1.00	1.00	1.00	1.00
6. You feel a stabbing sensation when there is pain:	1.00	1.00	1.00	1.00
7. You feel tense when there is pain:	1.00	1.00	1.00	1.00
8. The pain you feel is deep:	1.00	1.00	1.00	1.00
9. How bad has your pain been over the past week?	1.00	1.00	1.00	1.00
10. The pain you feel makes you dizzy:	1.00	1.00	1.00	1.00
11. The pain you present is annoying and makes you angry:	1.00	1.00	1.00	1.00
12. The pain you feel causes you anguish:	1.00	1.00	1.00	1.00
13. The pain you feel irritates you:	1.00	0.87	1.00	0.93
14. The pain you feel depresses you:	1.00	1.00	1.00	1.00
15. The pain you feels makes you desperate:	1.00	1.00	1.00	1.00
16. Pain interferes with your daily activities:	1.00	1.00	1.00	1.00
17. Pain has changed your ability to perform daily activities:	1.00	1.00	1.00	1.00
18. How much has your pain changed your ability to participate in social and recreational activities?	1.00	1.00	1.00	1.00
19. Pain has changed your ability to perform routine household activities:	1.00	1.00	1.00	1.00
20. You have been able to take care of the garden since you started to experience pain:	1.00	1.00	1.00	1.00
21. Have you gone for a walk since you started to experience pain?	1.00	1.00	1.00	1.00
22. Have you gone shopping since you started to experience pain?	1.00	1.00	1.00	1.00
23. Have you prepared any food since you started to experience pain?	1.00	1.00	1.00	1.00
24. Have you visited friends since you started to experience pain?	1.00	1.00	1.00	1.00
25. Have you visited family members since you started to experience pain?	1.00	1.00	1.00	1.00
26. You have helped clean the house since you started to experience pain?	1.00	1.00	1.00	1.00
27. You have bathed alone since you started to experience pain:	1.00	1.00	1.00	1.00
28. You have eaten on your own since you started to experience pain:	1.00	1.00	1.00	1.00
29. You have dressed yourself since you started to experience pain:	1.00	1.00	1.00	1.00
30. You have been able to go to the toilet on your own since you started to experience pain:	1.00	1.00	1.00	1.00

Source: Data of expert opinions obtained with Delphi technique, 2024.

During the third round, all the items met the CVR criteria, with a minimum of 0.87 and a maximum of 1.0, validating the 30 questions for integration into the scale as essential, useful, and necessary to measure pain in OAs and its influence on ADLs.

With the constructed scale, an exploratory factor analysis (EFA) was carried out to organize the items into dimensions. For this, a sample of 185 OAs who attend day homes was interviewed, of which 167 (90.3) were female and 18 (9.7%) were men; 37 (20%) were aged 60 to 65 years, 47 (25.3%) were aged 66 to 71 years, 30 (16.5%) were aged 78 to 83 years, 7 (3.7%) were aged 84 to 89 years, and 2 (1%) were aged 90 and over; and in terms of their marital status, 34 (18.4%) were single, 78 (42.2%) were married, 69 (37.3%) were widowed, and 4 (2.2%) were divorced, as shown in Table 6.

The exploratory factor analysis was carried out while taking into consideration the normality (distribution of variables), homoscedasticity (variability), and multicollinearity (correlation between variables) [21]. The Kaiser–Meyer–Olkin Measure of Sampling Adequacy test resulted in a value of 0.803, indicating a meritorious sample regarding the adequacy of the factor model. Bartlett’s sphericity test, an indicator that allows for the correlation between variables to be compared at a significance of <0.001, allowed the null hypothesis of the identity matrix to be rejected, which suggests multicollinearity between the variables. The exploratory factor analysis resulted in only 24 items being extracted from the latent root criterion (eigenvalues greater than 1), the percentage of variance, and the contrast of fall and were thus integrated into four factors: 1. pain and instrumental activities of daily living; 2. influence of pain on the affective sphere; 3. sensory perception of pain; and 4. autonomy in basic needs (Table 7). The factor loads need to be 0.50 or greater to be considered significant, because the larger the size of the factor load, the more important the load is for interpreting the matrix; therefore, six items were excluded because they had low factor loads, explained variances, and numbers of items in each factor.

The items grouped into factor 1, pain and the instrumental activities of daily living, are closely related to outside activities and other people; those in factor 2, the influence of pain in the affective sphere, are related to the emotions derived from a sensory response to pain; those in factor 3, the sensory perception of pain, are closely related to the sensations they perceive and the presence of pain; and finally, those in factor 4, the autonomy of basic needs, are related to the ability to autonomously address their needs. The commonality column shows the variance of each item explained by the factorial solution, where the greater the commonality, the greater the explained variance of the factor. With the results of this model, the total variance explained was 54.5%.

To analyze the reliability of each of the factors that make up the 24-item scale, McDonald’s Omega reliability coefficients were measured, giving the following results: factor 1, 0.919; factor 2, 0.881; factor 3, 0.810; and factor 4, 0.866 (Table 7). All were highly reliable, with reliability values greater than 0.70.

To validate the factors of the scale, a confirmatory factor analysis was performed, and to assess the adequacy of the favorable factor model and the need for sampling adjustment, the Kaiser–Meyer–Olkin Measure of Sampling Adequacy test was conducted, achieving a score of 0.803. Bartlett’s sphericity test, an indicator that allows for the correlations between the variables to be formally compared, had a significance of <0.001; the extraction method used was main axis factorization and rotation via Quartimax with Kaiser normalization in order to simplify the rows of the factor matrix. Table 8 shows the results, including the 24 items and four factors, taking 0.50 as the criteria for significance in the factor loads, as well as the McDonald’s Omega reliability coefficients obtained for each factor, showing high reliability.

## 4. Discussion

The SMPOA, which was designed to assess pain in OAs, is integrated with four factors: Factor 1 is pain and the instrumental activities of daily living (IADLs), which measures the influence of pain on daily life. This factor resembles the WHYMPI in that it is based on the cognitive theory of behavior. However, the difference is that factor 1 measures the pain-induced limitations in performing activities, such as shopping, preparing food, visiting relatives, participating in recreational activities, going for walks, and cleaning the house, while the PainDETEC instrument only measures neuropathic pain. Factor 2 takes into account the feelings of OAs and measures the affective sphere by evaluating the presence of irritability, anger, despair, and anguish, in the same way as in the one-dimensional numerical pain scale, which, however, only evaluates the intensity of the pain. Factor 3 includes the evaluation of the perception of pulsations, pinching sensations, tension, puncture, and depth perception, and factor 4 addresses aspects related to the influence of pain on performing activities of daily living, such as going to the bathroom, dressing, eating, and bathing.

The SMPOA was validated with statistical measurements and showed that in the presence of pain, the OAs experienced affective alterations: irritation, depression, despair, and anguish. As mentioned by De Andrés, Acuña, and Olivares (2014) [16], age, conditions that generate pain, and interactions with family and friends are associated with depressive symptoms, isolation, and functional alterations. Therefore, the affective dimension is related to the presence of pain.

Furthermore, the scale will help to assess the presence of pain and reach a diagnosis that contributes to timely intervention based on three dimensions: sensory intensity, affective intensity, and the influence of pain on ADLs. Therefore, this scale can feasibly be used in OAs who are cared for in day homes that provide primary care services, where care and interventions of this type are typically provided. This multidimensionality makes this scale different from the other scales that have been used in patients with arthritis to assess traumatology and the need for surgery.

The SMPOA measures pain in OAs in a multidimensional manner compared with existing scales that measure pain in only one dimension, thus addressing the pain-induced limitations in the performance of the ADLs. The language used in the scale can be understood by health personnel and was proposed by experts who care for and intervene in the care of OAs, such as gerontology professionals.

Finally, a limitation of the SMPOA is that it only measures pain in OAs because it was designed specifically for use with this study population; therefore, further measurements and validations in different age groups and by different health professionals are needed to explore pain characteristics in people with and without chronic disease and to strengthen the instrument.

## 5. Conclusions

The Delphi technique is useful and reliable in obtaining expert judgment on a specific topic, and Lawshe’s model is useful for validating the items created by the experts, including the sufficiency, clarity, coherence, and relevance of the items.

The SMPOA was integrated and validated with 24 items distributed into four factors and is an instrument that takes the changes associated with the aging process into consideration.

The scale can be feasibly used by professionals who provide primary care to OAs to assess the presence of pain and to provide care that helps maintain functionality in the lives of aging people. After further validation, it can be feasibly used to measure pain in other populations.

## Figures and Tables

**Table 1:** Evaluation of proposed items.

Category	Score	Indicator
SUFFICIENCY: the item belongs to a single dimension	1	The item is insufficient for measuring the dimension.
	2	The item measures some aspect of the dimension, but not completely.
	3	Some aspects must be increased for the item to fully evaluate the dimension.
	4	The item is sufficient.
CLARITY: the item is easy to read and understand	1	The item does not meet the criteria and is not clear.
	2	The item requires several modifications to its meaning or sentence structure.
	3	Specific modifications to the item are required.
	4	The item is clear and adequate.
COHERENCE: the item has a logical relationship with what is being measured	1	The item has no logical relationship to the dimension.
	2	The item has little coherence with what it intends to measure.
	3	The item has a moderate relationship with what it intends to measure.
	4	The item is logical and consistent with what it intends to measure.
RELEVANCE: the item is appropriate for the dimension	1	The item must be deleted for irrelevance.
	2	The item has little relevance but can be modified.
	3	The item is relevant but may need some modifications.
	4	The item is completely relevant.

Source: Escobar and Cuervo (2008, p. 37) [20].

**Table 2:** Professional characteristics of experts.

Number of Experts	Gender		Level of Education		Workplace		Experience	
	Female	Male	Bachelor’s Degree	Graduate	Hospital	Health Center	3 to 5 Years	More than 6 Years
15	11	4	15	4	2	13	7	8

Source: Data obtained from participants, 2024.

**Table 6:** Sociodemographic characteristics of OAs.

Sociodemographic Data		
Gender	N	Percentage
Female	167	90.3
Male	18	9.7
Total	185	100
Age		
60–65 years	37	20
66–71 years	47	25.3
72–77 years	62	33.5
78–83 years	30	16.5
84–89 years	7	3.7
90 years and over	2	1
Total	185	100
Marital status		
Single	34	18.4
Married	78	42.2
Widower	69	37.3
Divorced	4	2.2
Total	185	100

Source: SMPOA data, 2024.

**Table 7:** Exploratory factor analysis.

Item	Item	Factor 1	Factor 2	Factor 3	Factor 4	McDonald’s Omega	Communalities
	Factor 1: Pain and Instrumental Activities of Daily Living					0.919	
1	Have you gone shopping since you started to experience pain?	0.865					0.800
2	Have you visited friends since you started to experience pain?	0.850					0.795
3	Have you prepared any food since you started to experience pain?	0.830					0.786
4	You visit your relatives since you started to experience pain:	0.837					0.755
5	Have you gone for a walk since you started to experience pain?	0.782					0.678
6	You have helped with cleaning the house since you started to experience pain.	0.744					0.684
7	You have been able to take care of the garden since you started to experience pain:	0.698					0.563
8	How much has your pain changed your ability to participate in social and recreational activities?	−0.510					0.577
9	The pain has changed your ability to perform routine household activities.	−0.555					0.643
	Factor 2: Influence of Pain on the Affective Sphere					0.881	
10	The pain you feel irritates you:		0.870				0.808
11	The pain you feel makes you desperate:		0.815				0.810
12	The pain you present is annoying and makes you angry:		0.794				0.754
13	The pain you feel depresses you:		0.735				0.690
14	The pain you feel causes you anguish:		0.685				0.658
	Factor 3: Sensory Perception of Pain					0.810	
15	The pain you feel is deep:			0.748			0.737
16	How bad has your pain been over the past week?			0.706			0.652
17	You feel a pulsing sensation when there is pain:			0.701			0.613
18	You feel a pinching sensation when there is pain:			0.611			0.519
19	You feel tense when there is pain:			0.575			0.593
20	You feel a stabbing sensation when there is pain:			0.661			0.528
	Factor 4: Autonomy in Basic Needs					0.866	
21	You have been able to go to the toilet on your own since you started to experience pain:				0.830		0.743
22	You have dressed yourself since you started to experience pain:				0.820		0.785
23	Has eaten on their own since starting to experience pain:				0.799		0.743
24	Has bathed on their own since they started to experience pain:				0.794		0.778
	Extraction Method: Principal Component Analysis. Rotation Method: Quartimax with Kaiser Normalization.						

**Table 8:** Confirmatory factor analysis.

Item	Item	Factor 1	Factor 2	Factor 3	Factor 4	McDonald’s Omega	Communalities
	Factor 1: Pain and Instrumental Activities of Daily Living					0.919	
1	Have you visited friends since you started to experience pain?	0.825					0.706
2	Have you gone shopping since you started to experience pain?	0.865					0.796
3	You have visited your relatives since you started to experience pain:	0.820					0.690
4	Have you prepared any food since you started to experience pain?	0.769					0.659
5	Have you gone for a walk since you started to experience pain?	0.737					0.576
6	Has helped with cleaning the house since they started to experience pain:	0.731					0.593
7	You have been able to take care of the garden since you started to experience pain:	0.648					0.466
8	How much has your pain changed your ability to participate in social and recreational activities?	−0.511					0.392
9	The pain has changed your ability to perform routine household activities:	−0.584					0.435
	Factor 2: Influence of Pain on the Affective Sphere					0.881	
10	The pain you feel irritates you:		0.866				0.826
11	The pain you present is annoying and makes you angry:		0.744				0.700
12	The pain you feel makes you desperate		0.738				0.718
13	The pain you feel depresses you:		0.587				0.537
14	The pain you feel causes you anguish:		0.521				0.426
	Factor 3: Sensory Perception of Pain					0.810	
15	The pain you feel is deep:			0.721			0.594
16	You feel a pulsing sensation when there is pain:			0.686			0.495
17	How bad has your pain been over the past week?			0.668			0.535
18	You feel a stabbing sensation when there is pain:			0.634			0.422
19	You feel a pinching sensation when there is pain:			0.526			0.295
20	You feel tense when there is pain:			0.469			0.243
	Factor 4: Autonomy in Basic Needs					0.866	
21	You have been able to go to the toilet on your own since you started to experience pain:				0.788		0.665
22	You have dressed yourself since you started to experience pain:				0.830		0.751
23	Has eaten on their own since starting to experience pain:				0.668		0.499
24	Has bathed on their own since they started to experience pain:				0.760		0.704
	Extraction Method: Primary Axis Factorization Rotation Method: Quartimax with Kaiser Normalization						

## Data Availability

Data are contained within the article.

## References

[1] García-Andreu J. (2017). Basic Management of Acute and Chronic Pain. Anesth. Mex..

[2] Raja S.N., Carr D.B., Cohen M., Finnerup N.B., Flor H., Gibson S., Keefe F.J., Mogil J.S., Ringkamp M., Sluka K.A. (2020). The revised International Association for the Study of Pain definition of pain: Concepts, challenges, and compromises. Pain.

[3] Guevara-López U. (2010). Pain of the Musculoskeletal System. Mex. J. Anethesis.

[4] Pérez-Castañeda T. (2012). Pathophysiology of Acute Pain: Cardiovascular, Respiratory, and Other Systems and Organ Disorders. Cuba J. Anesthesiol. Resusc..

[5] Welsh T.P., Yang A.E., Makris U.E. (2020). Musculoskeletal Pain in Older Adults: A Clinical Review. Med. Clin. N. Am..

[6] Corsi N., Roberto A., Cortesi L., Nobili A., Mannucci P.M., Corli O. (2018). Prevalence, characteristics and treatment of chronic pain in elderly patients hospitalized in internal medicine wards. Eur. J. Intern. Med..

[7] González-Estavillo A.C., Jiménez-Ramos A., Rojas-Zarco E.M., Velasco-Sordo L.R., Chávez-Ramírez M.A., Coronado-Ávila S.A. (2018). Correlation between One-Dimensional Scales Used in the Measurement of Postoperative Pain. Mex. J. Anesthesiol..

[8] Romero G., Romero J., Abascal F., Carrillo S. (2002). Pain Measurement: An Update. Rev. Med. Integral.

[9] León-Mateos L. (2005). Dimensions of pain in patients with temporary disability of musculoskeletal origin. J. Psychol. Psychopedagogy.

[10] Herrero M.T.V., Bueno S.D., Moyá F.B., de la Torre M.V.R.I., García L.C. (2018). Valuation of Pain. Comparative Review of Scales and Questionnaires. J. Span. Soc. Pain.

[11] National Population Council (2021). International Day of Older Persons, gob.mx. https://www.gob.mx/conapo/articulos/dia-internacional-de-las-personas-de-edad-284170?idiom=es.

[12] World Health Organization (2022). Ageing and Health. https://www.who.int/es/news-room/fact-sheets/detail/ageing-and-health.

[13] Mexican Institute of Social Security (2013). Clinical Practice Guideline Management of Geriatric Syndromes Associated with Postoperative Complications. https://www.imss.gob.mx/sites/all/statics/guiasclinicas/612GER.pdf.

[14] National Institute of Statistics and Geography (2019). Statistics on the International Day of Persons with Disabilities. https://www.inegi.org.mx/contenidos/saladeprensa/aproposito/2019/Discapacidad2019_Nal.pdf.

[15] Alvarado García A.M., Salazar Maya A.M. (2016). Uncovering the Feelings and Behaviors Experienced by Older Adults with Benign Chronic Pain. Gerokomos.

[16] DeAndrés J., Acuña J.P., Olivares S.A. (2014). Pain in the Elderly Patient. Revista Médica Clínica Las Condes.

[17] Varela M., Díaz L., García R. (2023). Description and Uses of the Delphi Method in Health Research. Med. Res. Educ..

[18] Acevedo González J.C. (2013). Ronald Melzack and Patrick Wall. The gate theory. Beyond the scientific concept, two worlds scientists dedicated to the understanding of pain. J. Span. Soc. Pain.

[19] Zaneti Díaz P., Martínez Triana R., Castillo González D. (2020). Pain: Some Psychological Criteria. Cuba J. Hematol. Immunol. Hemotherapy.

[20] Escobar Pérez J., Cuervo Martínez Á. (2008). Content Validity and Expert Judgment: An Approach to Its Use. Adv. Meas..

[21] Hair J.F., Anderson R.E., Tatham R.L., Black W.C. (1999). Multivariate Data Analysis.

